# Under-five mortality in The Gambia: Comparison of the results of the first demographic and health survey with those from existing inquiries

**DOI:** 10.1371/journal.pone.0219919

**Published:** 2019-07-23

**Authors:** Anne J. Rerimoi, Momodou Jasseh, Schadrac C. Agbla, Georges Reniers, Anna Roca, Ian M. Timæus

**Affiliations:** 1 Department of Population Health, London School of Hygiene and Tropical Medicine (LSHTM), London, United Kingdom; 2 Disease Control and Elimination Theme, Medical Research Council Unit The Gambia at LSHTM, Fajara, The Gambia; 3 Department of Medical Statistics, London School of Hygiene and Tropical Medicine (LSHTM), London, United Kingdom; University of Louvain, BELGIUM

## Abstract

**Background:**

In The Gambia, national estimates of under-five mortality (U5M) were from censuses and multiple indicator cluster surveys (MICS). The country’s first demographic and health survey (DHS) conducted in 2013 provided empirical disaggregated national estimates of neonatal, post-neonatal and child mortality trends.

**Objective:**

To assess the consistency and accuracy of the estimates of U5M from the existing data sources and its age-specific components in rural Gambia and produce reliable up-to-date estimates.

**Methods:**

Available national data on under-five mortality from 2000 onwards were extracted. Additionally, data from two DHS regions were compared to those from two health and demographic surveillance systems (HDSS) located within them. Indirect and direct estimates from the data were compared and flexible parametric survival methods used to predict mortality rates for all empirical data points up to 2015.

**Findings:**

Internal consistency checks on data quality for indirect estimation of U5M suggest that the data were plausible at national level once information from women aged 15–19 years was excluded. The DHS and HDSS data used to make direct U5M estimates were plausible, however HDSS data were of better quality. For 2009–2013, the DHS estimates agreed well with the 2013 census and 2010 MICS reports of U5M but was less accurate about the early births of older women. The most recent estimates from the 2013 DHS, which refer to 2011–12, are an U5M rate of 54/1000 livebirths (95% CI: 43–64) and a neonatal mortality rate of 21/1000 livebirths (95% CI: 15–27), contributing almost 40% of U5M in The Gambia. The DHS showed that for the decade prior to the survey, child mortality dropped by 55% and neonatal mortality by 31%. This indicates the importance of neonatal mortality in The Gambia, and the need to focus on neonatal survival, while maintaining currently successful strategies to further reduce U5M.

## Introduction

Under-five mortality is an important indicator of a population’s health and is strongly related to structural factors such as economic status, social wellbeing and environmental factors [1–3]. Sub-Saharan Africa (SSA) has the highest under-five mortality rates (U5MR) of any world region, with West and Central Africa worst affected. Almost half of under-five deaths (44%) occur during the first month of life and two-thirds of these neonatal deaths occur during the first few days of life [4]. Global targets to reduce under-five mortality by two-thirds between 1990 and 2015 were set as part of the Millennium Development Goals. Although this target was not achieved by 2015, global under-five mortality was halved [2]; and some African countries registered significant gains in child survival [4, 5]. Consequently, the new Sustainable Development Goals (SDGs) have set a target of reducing neonatal (NMR) and under-five mortality rates by 2030 to 12 and 25 deaths per 1000 livebirths, respectively [6]. The monitoring and evaluation of these goals require accurate statistics for mortality measurement.

Measuring mortality is straightforward when vital registration is complete. In SSA, vital registration is not fully functional in most countries [7–9], leaving the region dependent on alternative sources for estimating national mortality indices. These include national population censuses, Demographic and Health Surveys (DHS) and Multiple Indicator Cluster Surveys (MICS). Because of the relatively high cost and long periodicity of national censuses, DHS has become the main source for estimates of childhood mortality for use in national development planning and policy formulation [10]. One advantage of the DHS over other sources of childhood mortality data is the detailed birth histories it collects. These data can be used to calculate the different age-specific indicators of childhood mortality, namely: neonatal (< 28 days), post-neonatal (1–11 months), child (1–4 years) and under-5 mortality rates.

Prior to its first DHS in 2013, The Gambia was one of very few continental sub-Saharan African countries that still depended solely on decennial censuses and MICS as sources of childhood mortality data. Indirect estimates from successive decennial censuses since 1973, as well as three MICS enquiries between 2000 and 2010, document substantial decline in under-five mortality over the last four decades [11–16]. Additionally, longitudinal data from a health and demographic surveillance system (HDSS) suggest that U5MR fell below the MDG 4 goal seven years early [17]. To date, no attempt has been made to systematically compare childhood mortality estimates derived from each of these sources.

The release of complete birth history data from the 2013 DHS therefore provides an opportunity to update and reassess mortality information from all available data sources. First, consistency checks are used to evaluate the quality of the DHS against existing Gambian mortality data, that is indirect estimates of under-five mortality from the MICS and the censuses and direct estimates of under-five mortality and its constituents obtained from the HDSS. Then, new estimates are presented of under-five mortality and its components based on the conclusions reached about data quality and comparability of the DHS to existing sources.

## Methods

### Study setting

The Gambia is one of the smallest continental African countries. It is located in the middle of the bulge of the West African coast; and bound to the north, east and south by Senegal, and to the west by the Atlantic Ocean. With a total population of 1.85 million as at the 2013 national census[13], the country is divided for administrative purposes into two urban municipalities and five rural local government areas (LGAs). Two of these rural LGAs, Kerewan and Basse, host the Farafenni and Basse HDSS sites, respectively.

### Ethics statement

Farafenni and Basse HDSS have the joint MRC/ Gambia Government Ethics committee approval to conduct continued surveillance. No ethical approval was required to use the data from DHS and MICs which are available on request having received approval to be conducted by the respective Institutional Review Boards and the Gambia Government.

### Data sources

In addition to the 2013 DHS, five independent enquiries that collected childhood mortality data have been conducted by the Gambia Bureau of Statistics (GBoS) since 2000, which is the starting period for this study. These data comprise two sets of summary birth histories from the 2003 and 2013 national censuses, and three sets of summary birth histories from the nationally representative MICS 2000, 2005/6 and 2010. Whilst the DHS collected similar summary data, it also obtained data on person-time at risk and age at death by collecting full birth histories, thus enabling the *direct estimation* of mortality rates [18]. In contrast, the censuses and MICS asked women of reproductive age about their numbers of children ever born and surviving, thereby providing the key information required for *indirect estimation* of childhood mortality [19]. Regional data were extracted from the DHS for Kerewan and Basse LGAs. The indirect estimation technique uses information that indirectly represents the parameter being measured. For example, the proportion dead of children ever-born is influenced by both existing mortality conditions and maternal age, among other variables, meaning that it is not a pure mortality estimate. In contrast, direct estimation methods utilize the number of deaths as the numerator divided by a defined population as the denominator over specific time periods thus directly estimating the parameters of interest.

The individual-level data required to measure childhood mortality directly were also extracted from the Farafenni and Basse health and demographic surveillance systems. HDSS and DHS data were compared for the periods 2003–4, 2005–7, 2009–10 and 2011–12 due to availability of HDSS data that were reliable. The Farafenni HDSS has been in operation since 1981 and covered a population of about 55,000 by December 2015. It is described in detail elsewhere[20]. The Basse HDSS started in 2007 and currently follows up about 180,000 people. It adopts the same procedures that apply in the FHDSS. In combination, the two HDSSs follow up about 13% of the Gambian population. Heads of household, or appropriate representatives, are interviewed at least once every four months about births, deaths, migrations and pregnancies that occurred in the period since the last visit. When pregnancies are identified, they are followed up in subsequent rounds of update to establish their outcomes[20].

### Data quality assessment

The accuracy of indirect estimates of childhood mortality depends, among other factors, on the quality of the data relating to children ever born, proportions dead, and the fertility experience of mothers by age group [21]. Consistency checks were therefore applied to ascertain the plausibility of the sex ratio at birth (SRAB) of children ever born; the distribution of proportions dead of children ever born by age of mother; and the trend of average parities by age of mothers. As the natural SRAB is about 1.05, ranging between 1.03 and 1.07, male children born for every female in most populations around the world, the SRAB was examined for children ever born reported in the MICS, census and DHS datasets.

The quality of data used for direct estimation of mortality is usually assessed based on completeness of reporting of dates of births and deaths, and tests for omissions of births or deaths, birth displacement, age heaping and date preference. Assessment of these for the DHS data yielded similar outcomes as indicated in the report, i.e. good quality of date reporting with less than 2% missing overall [12]. There was heaping of reported ages at death at 6 and 12 months, with only the latter likely to affect infant mortality estimates. The data from the two HDSS sites had complete dates of birth and death. However, inspection of the distribution of reported day of birth and death in the Basse HDSS data revealed that the first day of the month was by far the most frequently reported, suggesting that it was recorded when the actual day could not be ascertained (see S1 File). To minimise the potential impact on the estimates that this issue might have had as a result of displacing neonatal deaths to the post-neonatal period, dates for births and deaths that occurred on the first day of the month were randomly imputed while preserving the reported month.

### Estimation methods

Using the individual-level data from the HDSS, mortality rates were calculated directly for each period and year using Kaplan–Meier failure probabilities for each of the following age groups: <28 days for neonatal mortality; 28 days to <1 year for post-neonatal mortality; 1–4 years for child mortality; and <5 years for under-5 mortality. The HDSS data were extracted from 1990 to 2014 for Farafenni (excluding 2008/2009) and from 2009 to 2014 for Basse. The year 1990 was chosen to reflect MDG trends in U5MR in view of good quality prospectively collected data from Farafenni HDSS. However, 2008/9 was excluded as the Farafenni field station was temporarily closed from February 2008 to March 2009 and the quality of data for this period collected retrospectively was not reliable [20]. Data from Basse HDSS in 2007/8, when surveillance began was unreliable, but markedly improved by 2009 and thus analysis was done from 2009 onwards (see S2 File). The 2013 Gambia DHS was used to estimate both national mortality and mortality in the Kerewan and Basse regions using the synthetic cohort probabilities method employed by demographic health surveys [22]. For more accurate and conservative standard errors for the DHS data, clustering was accounted for, and 1000 sample replications used to generate bootstrapped standard errors and 95% confidence intervals for the mortality rates [23].

The 2003 and 2013 censuses and the 2000, 2005/6 and 2010 MICS were analysed using the indirect method to estimate under-five mortality from the means of children ever-born and surviving for five-year maternal age groups[24]. The method incorporates assumptions about the age pattern of mortality. Princeton South model life tables were used to produce the estimates for comparison purposes, as these were utilized in previous analyses of the MICS and censuses. We do not report on rates generated from the 15–19 age group [21, 24].

Indirect estimates from the DHS were compared to estimates obtained from these data sources in order to make a pure comparison of the completeness of death reporting in each inquiry. Having done this, the rest of the paper focuses on direct estimates from the DHS which, on the assumption that reporting of ages and dates is accurate, allows us to estimate the age pattern of mortality from the data.

For the direct estimates only, we used flexible parametric survival methods to generate smooth mortality trends by calendar year [25]. As more flexible models did not converge, the model was limited to one knot for neonatal and post-neonatal mortality, and to two for child mortality. Both smoothed and direct non-parametric mortality rates were plotted.

## Results

### Data quality assessment

**Fig 1:**
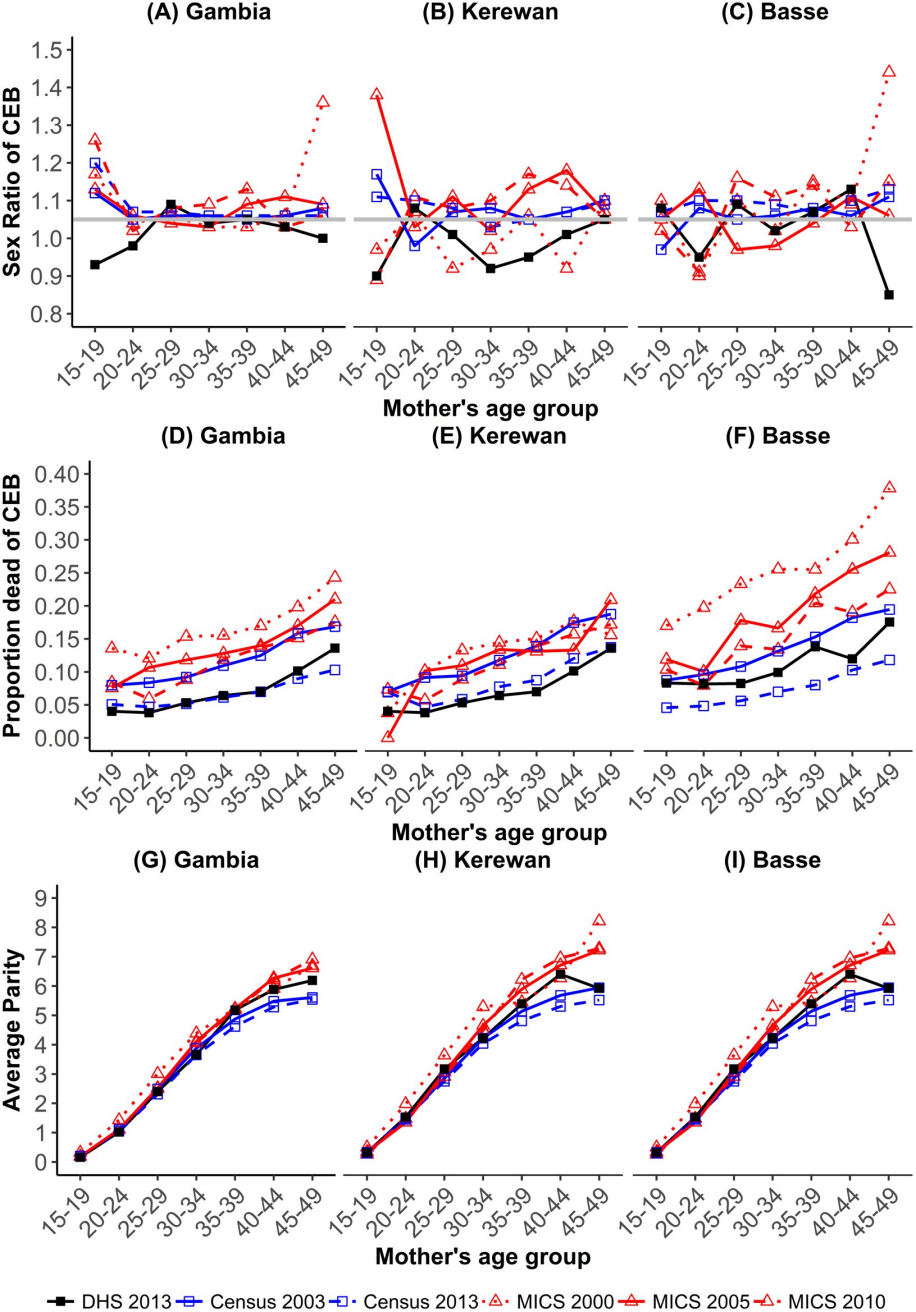
Assessment of the data on children ever-born (CEB) and dead children MICS 2000,2005/6 & 2010, census 2003,2013 and DHS.

The results of data quality checks on MICS, censuses and DHS by means of sex ratio of children ever born, proportion dead of children ever born and average parity by mother at birth are presented in Fig 1.

The sex ratio of children ever born at national level converged around 1.05 for older mothers, being erratic for the 15-19-year age group. In the regions, after excluding extremes of age (<20 and >40 years of age), the sex ratio at birth did not significantly diverge from 1.05 (for all p>0.05). Although Kerewan DHS reported a SRAB less than 1.05 for all age groups, it was not far off from the expected SRAB.

The expected pattern of an increase in proportions dead of children ever born with maternal age was observed with few exceptions. At national level, proportions dead of children ever born were higher for the 15-19-year age group than the 20-24-year age group for the MICS in 2000 and 2010. In Basse, the DHS and MICS showed instances of lower proportions dead of children ever born in older age groups. For example, women aged 35–39 years had a higher proportion of dead children than those aged 40–44 years. Such fluctuations were also seen in the MICS data, occurring most often in the MICS in 2010. In Kerewan, this was only seen in the MICS conducted in 2005/6, with the remaining data sources depicting the expected pattern once the 15-19-year age group was excluded.

We observed expected trends in average parity per woman at national level from the DHS, while regionally, the 45-49-year age group registered slightly lower average parities than expected. Overall, the MICS had slightly higher average parity and higher proportions dead of children than other sources, particularly the MICS in 2000 in Basse.

### Consistency of estimates of under-five mortality from different inquiries

**Fig 2:**
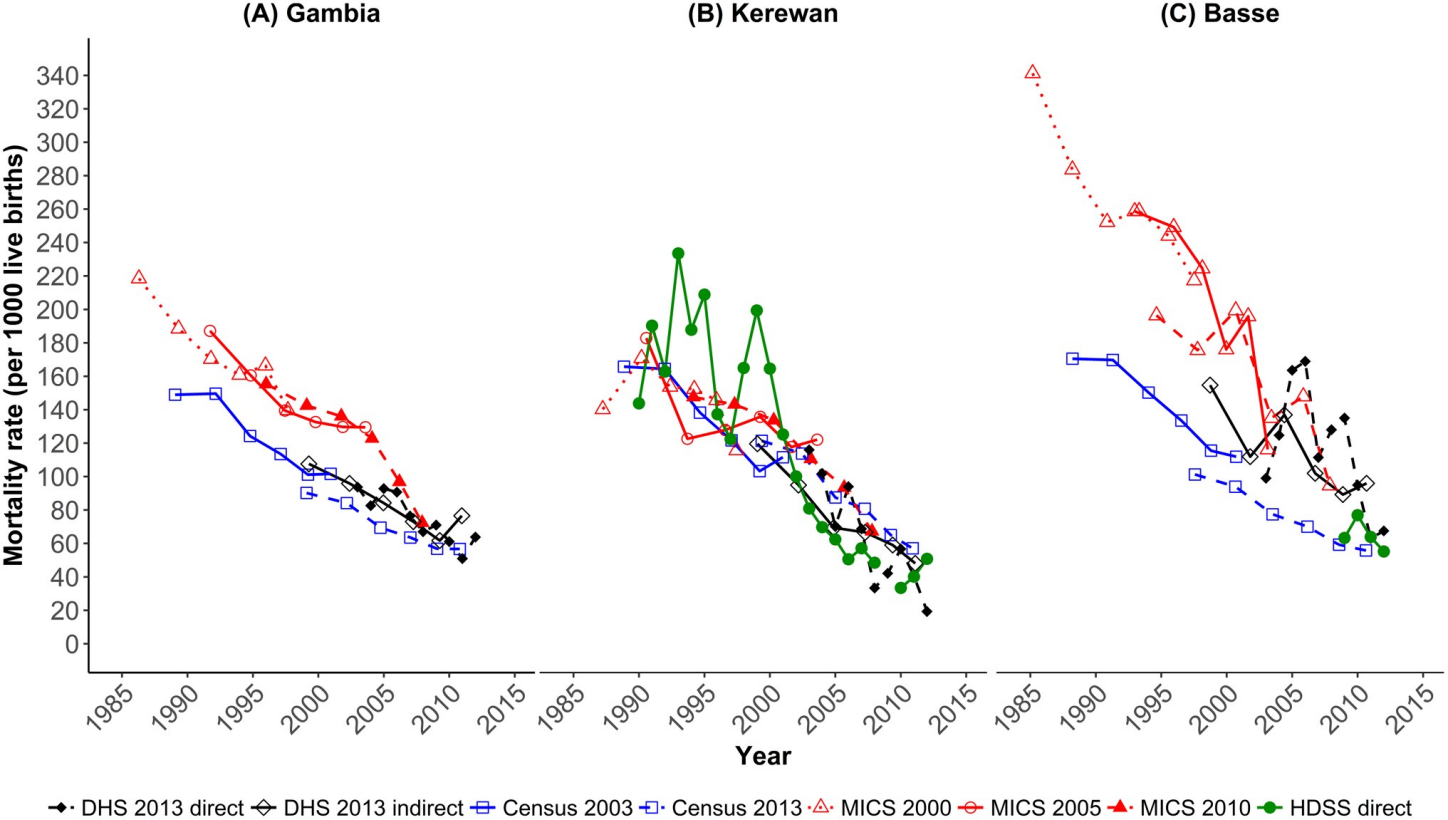
Under-five mortality estimates for The Gambia, Kerewan and Basse regions using data from 2003 and 2013 census, MICS 2000, 2005/6 and 2010, DHS 2013 and HDSS located within Basse and Kerewan regions.

Direct and indirect estimates of under-five mortality at the national and selected regional levels are presented graphically in Fig 2 for all eight data sources (3 MICS, 2 Censuses, 1 DHS and 2 HDSS). All sources depicted a decrease in under-five mortality in The Gambia.

#### National level

At the national level (see Fig 2, Panel A), the estimates of under-five mortality from the two censuses are consistent with each other and with the DHS, while the estimates from the MICS are much higher, especially before 2005. The estimated under-five mortality rates in 1999, for example, were 142, 90 and 108 per 1000 livebirths according to the 2010 MICS, the 2013 census and the 2013 DHS respectively, a gap of up to 50/1000 livebirths. The most recent estimates from the latest MICS survey, referring to 2006 to 2008, however, converge with those from the DHS (72/1000 livebirths and 73/1000 livebirths, respectively), although the census estimate was slightly lower (64/1000 livebirths). This discrepancy persists in the period 2009–2011, with DHS estimating under-five mortality at 69/1000 livebirths and the census at 57/1000 livebirths. The DHS direct and indirect estimates followed parallel trends.

**Fig 3:**
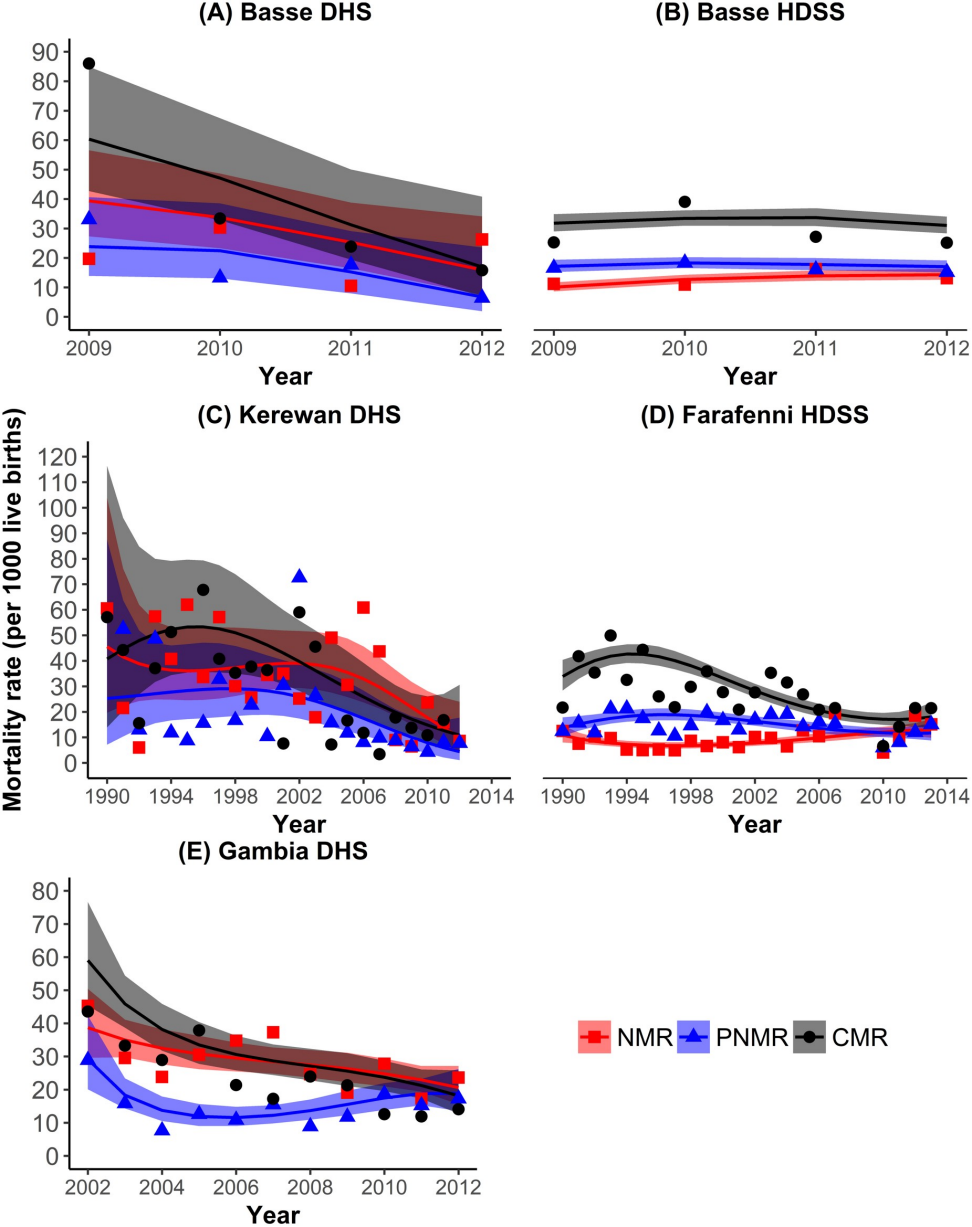
Direct estimates of neonatal, post-neonatal and child mortality rates in Basse and Farafenni HDSS, Basse and Kerewan regions and the Gambia.

Having ascertained that the most recent indirect estimates from the DHS were broadly comparable to those from other data sources, we calculated direct estimates from the DHS of the age-specific components of under-five mortality (details on point estimates and 95% CI from direct estimation of DHS data are presented in Table 1 and depicted in Fig 3, Panel E). The most recent estimates from the 2013 DHS, which refer to 2011–12, are a U5MR of 54/1000 livebirths (95% CI: 43–64) and an NMR of 21/1000 livebirths (95% CI: 15–27), with the latter contributing almost 40% of under-five mortality in The Gambia. The DHS additionally showed that for the ten years prior to the survey, child mortality dropped by 55% and neonatal mortality by 31% (Table 1).

**Table 1:** Neonatal, post-neonatal, child and under-five mortality rates and rate differences (95% CI) for HDSS and DHS regions and periods covering 2003–2012 in The Gambia.

**The Gambia**	**NMR**	**(95% CI)**		**PNMR**	**(95% CI)**		**CMR**	**(95% CI)**		**U5MR**	**(95% CI)**	
2003–4	30∙2	(20∙7–39∙7)		16∙9	(10∙7–23∙0)		36∙9	(27∙4–46∙3)		82∙2	(67∙4–97∙1)	
2005–7	34∙6	(27∙1–42.0)		13∙2	(8∙5–17∙9)		28∙0	(20∙1–35∙9)		74.4	(62∙4–86∙5)	
2009–10	24∙4	(17∙4–31∙5)		9∙7	(5∙1–14∙2)		25∙3	(16∙1–34∙5)		58.5	(47∙3–69∙7)	
2011–12	20∙9	(14∙5–27∙2)		16∙5	(7∙4–25.6)		16∙7	(11∙3–22∙1)		53.5	(43∙0–64.0)	
	**NMR**			**PNMR**			**CMR**			**U5MR**		
	**DHS** **(95% CI)**	**HDSS** **(95% CI)**	**Rate Difference**	**DHS** **(95% CI)**	**HDSS (95% CI)**	**Rate Difference**	**DHS** **(95% CI)**	**HDSS** **(95% CI)**	**Rate Difference**	**DHS** **(95% CI)**	**HDSS** **(95% CI)**	**Rate Difference**
			**(95% CI)**			**(95% CI)**			**(95% CI)**			**(95% CI)**
**Basse**												
2003–4	37.7 (12.1–63.2)			16.2 (1.1–31.2)			59.1 (31.9–86.2)			109.7 (78.9–140.5)		
2005–7	54.9 (31.9–77.9)			16.3 (3.5–32.6)			56.9 (27.1–86.6)			124 (82.8–165.2)		
2009–10	26.1 (12.1–40.2)	13.4 (11.7–15.7)	12.7 (-1.5;27)	18.9 (3.2–34.6)	20.7 (18.5–23.4)	-1.8 (-17.7;14.1)	56.2 (31.7–80.7)	37.4 (34.3–40.9)	18.8 (-5.9;43.5)	98.8 (72.2–125.3)	70 (66–74.8)	28.8 (1.8;55.7)
2011–12	16.4 (5.4–27.5)	15.9 (14.1–18.2)	0.5 (-10.6;11.8)	21.9 (9.0–34.8)	16.8 (14.8–19.1)	5.1 (-8;18.1)	21.2 (5.9–36.6)	27.8 (25.1–30.7)	-6.6 (-22.1;9.0)	58.7 (37.6–79.9)	59.4 (55.7–63.5)	-0.7 (-22.1;20.8)
**Kerewan**												
2003–4	38.0 (15.4–60.7)	8.4 (5.7–12.4)	29.6 (6.7;52.5)	20.5 (3.3–37.7)	25.6 (20.5–32.0)	-5.1 (-23.1;13.1)	42.5 (20.5–64.5)	43.0 (36.3–50.9)	-0.5 (-23.7;22.7)	98.6 (61.2–135.5)	75.4 (66.5–85.3)	23.2 (-15;61.4)
2005–7	43.5 (24.9–62.1)	12.1 (9.4–15.5)	31.4 (12.5;50.2)	14.8 (4.7–25.0)	18 (14.7–22.1)	-3.2 (-14;7.6)	13.7 (3.6–23.8)	27.7 (23.4–32.8)	-14 (-25.1; -2.9)	71.2 (47–95.3)	56.7 (50.6–63.5)	14.5 (-10.6;39.5)
2009–10	20.5 (4.0–37.0)	2.7 (1.4–5.2)	17.8 (1.2;34.3)	2.1 (2.0–6.2)	8.3 (5.8–11.9)	-6.2 (-11.3;-1.1)	22.2 (10.1–34.3)	21.2 (17–26.5)	1 (-11.9;14)	44.3 (23.4–65.2)	32 (26.7–38.3	12.3 (-9.3;34)
2011–12	9.1 (0.3–17.8)	12.7 (9.6–16.8)	-3.6 (-13;5.8)	3.1 (1.3–7.4)	12 (8.9–16)	-8.9 (-14.5; -3.4)	13.3 (2.2–24.3)	21.5 (17.3–26.7)	-8.2 (-20.2;3.8)	25.3 (10.4–40.1)	45.7 (39.5–52.9)	-20.4 (-36.7;-4.1)

NMR–Neonatal Mortality Rate, PNMR-Post-neonatal mortality rate, CMR- Child mortality rate, U5MR-Under-five mortality rate

#### Regional level

Panels B and C of Fig 2 present direct and indirect estimates of under-five mortality in Kerewan and Basse, the regions in which the two HDSSs are located. The estimates for Kerewan show a high degree of congruence between the MICS, the DHS and the census especially in the most recent five-year period. Direct estimates from Farafenni HDSS which is located within Kerewan region are also plotted and are congruent with the indirect data sources barring fluctuations in the early 1990s.

In Basse region, the census estimates are consistently lower than those from the other data sources, with a difference in point estimates of over 100/1000 livebirths in earlier periods. The 2000 MICS estimated U5MR at 284/1000 livebirths in 1988, while the 2003 census-based estimate was 170/1000 livebirths (Fig 2 Panel C). By 2007, the 2010 MICS estimate of the U5MR was 95/1000 livebirths; that from the DHS was 96/1000 livebirths; and the census reported 65/1000 livebirths. The MICS and DHS estimates are similar to each other from 2004 onwards. The direct Basse HDSS estimates are nearly congruent with the indirect estimates.

### Consistency of direct estimates of under-five mortality in the DHS and HDSS

To further evaluate the DHS data, consistency checks were performed by comparing disaggregated direct estimates of under-five mortality calculated from it with those from the Farafenni and Basse HDSS. The results are shown in Table 1 and depicted in Fig 3 (Panels A-D). Flexible parametric survival methods were applied to predict trends and confidence intervals for NMR, PNMR and CMR and plotted alongside non-parametric point estimates of the mortality rates in the HDSS and DHS regions. The DHS reported steeper declines in U5MR than the HDSS. According to the DHS, U5MR fell by 41% in the years 2009–2012 in Basse and by 74% in Kerewan from 2003–2012, with most of the decline being in child and post-neonatal deaths in the latter region. For the same periods, Basse HDSS reported a 15% reduction in the U5MR, and a 44% reduction in Farafenni. Estimates of neonatal, post-neonatal, child and under-five mortality rates between 2009 and 2012 in Basse DHS and HDSS were similar (p>0.05 for all), the only significant difference being higher under-five mortality in 2009–10 in the DHS.

On the other hand, prior to 2011, Kerewan DHS reported significantly higher estimates of neonatal mortality than Farafenni HDSS. Post-neonatal mortality rates were similar except in 2009–10 when they were higher in the HDSS. Regarding child mortality, there was no difference in estimates except in 2005–7 where the HDSS reported significantly higher rates (p = 0.04). There was no difference between the under-five mortality rates in Kerewan and Farafenni over the period 2003–2010. Under-five and post-neonatal mortality estimates for 2011–12 are significantly higher in Farafenni HDSS than in Kerewan DHS. Regarding Kerewan DHS, the most recent mortality estimates (2011–12), particularly in the post-neonatal period are uncharacteristically low, but the sample size on which it is based is quite small.

Regarding age patterns of mortality, child mortality shows the largest drop, starting earlier in Kerewan region. In both regions, neonatal mortality in DHS and HDSS in the most recent five years was less than 20/1000 livebirths. For the period 2011–12, NMR by data source were: Kerewan DHS NMR 9.1 (95% CI: 3.6–17.8); Basse DHS 16.4 (95% CI: 5.4–27.5); Farafenni HDSS 12.7 (95% CI: 9.6–16.8); and Basse HDSS 15.9 (95% CI: 14–18.1) per 1000 livebirths (Table 1).

## Discussion

This paper aimed to assess the data quality of the first Gambian DHS through consistency checks and compare estimates derived from it with those from the MICS and censuses in The Gambia. The internal consistency checks on the quality of data from six sources (MICS 2000, 2005 and 2010; Census 2003 and 2013 and DHS 2013) suggest that the data were plausible at national level once data obtained from women aged 15–19 years was excluded. These estimates, based on the 15–19 age group, are probably biased upward as very young mothers experience higher child mortality than older women [24]. Additionally, a study conducted to evaluate the accuracy of estimates from responses by 15–19 year olds and 20–24 year olds in demographic health surveys conducted consecutively showed that there were marked differences in reporting by age group [26]. Women aged 15–19 reported less accurately about their first births and first marriage, potentially due to social desirability and prevailing cultural, social and legal circumstances. Thus, estimates based on reports by younger women should be viewed with caution, particularly in sub-Saharan Africa [24, 26]. However, these consistency checks do no more than establish that the data are not grossly deficient as they only test for reliability of the data based on expected patterns [24]. The regional data on average parities showed minor deviations from expected patterns, and in Basse inconsistencies in proportions dead of children ever born were observed, particularly in the MICS data.

We also assessed the consistency of the estimates from the different inquiries. The MICS under-five mortality estimates for more than five years prior to the survey were higher than those from the DHS, while the national census estimates were lower than those from other data sources[16]. The discrepancy between the MICS and DHS data of up to 50/1000 livebirths cannot be fully explained by misreporting of stillbirths, estimated at 11/1000 births for five years prior to the Gambia DHS [12], as neonatal deaths. It is also unlikely that women reported deaths that did not occur in the MICS. This suggests that DHS data quality declines for earlier periods, that is, the data supplied by older women in the DHS are deficient, and significantly underestimate mortality. The low DHS mortality estimates in earlier periods should therefore be interpreted cautiously as dead children are likely to have been omitted. The stillbirth rate in the DHS report is also low, which could be a result of either misclassification of stillbirths as livebirths or more plausibly, the undercounting of adverse pregnancy outcomes. According to the 2013 Gambia census, the maternal mortality rate was 861/100,000 livebirths in the year prior to the census [27]. As stillbirths and maternal deaths are interlinked, it is likely that the low stillbirth rates are not reflective of the true situation [28]. The undercount of stillbirths is not unique to the DHS and is likely to occur in data collection methods that do not follow up pregnancies as a cohort. In addition, MICS and DHS have been found to have birth transference for the most recent five-year period which could contribute to underestimation of mortality [29]. The Gambia 2010 MICS despite improvement from previous surveys still had evidence of transference, however this was more evident with the 2013 Gambia DHS [12, 16]. At the regional level, census estimates for Basse were markedly lower than those from the other data sources, even in comparison to the lower census estimates at national level, pointing to inaccuracy of census data on under-five mortality in the Basse region.

A comparison of direct and indirect estimates using DHS data suggested that the assumptions involved in making indirect estimates did not strongly distort the estimates and validates the choice of the Princeton South model. This in turn implies that the indirect estimates from MICS are indicative of the overall extent of decline in under-five mortality in The Gambia since the 1990s. Consistency of direct and indirect estimates also implies that no evidence exists of gross errors in reporting of dates and ages at death in the DHS in the last decade [29]. Because deaths of children of a cohort of women do not all occur at the same time but are spread over several years, the indirect estimates tended to smooth out the fluctuations in the direct estimates. According to Silva, direct and indirect methods of estimation for under-five mortality can be interchanged for populations that did not experience shocks in mortality and fertility for the five-year age groups between 25–39 years [29].

The DHS and HDSS data used to derive direct under-five mortality estimates were of good quality for the recent past. HDSS quality was improved when imputation of data was done to counter date preference seen in Basse HDSS. Consistency of data for direct estimation was checked by comparing DHS and HDSS estimates. In Basse, the DHS and HDSS estimates of neonatal, post-neonatal and child mortality for 2009–2012 were similar. The estimates of under-five mortality from the Farafenni HDSS and for Kerewan region in the DHS are also comparable, but differences existed in those of neonatal and post-neonatal mortality. Using 2010 as the benchmark for the differences, we see that prior to 2010, the DHS for Kerewan reported significantly higher neonatal mortality estimates, while after 2010, the Farafenni HDSS reported significantly higher post-neonatal mortality rates. The low HDSS 2009–10 estimate of NMR could be due to undercounting following resumption of surveillance activities after the closure of the MRC Field Site [20]. In particular, lower neonatal mortality rates in the HDSS could be a result of early deaths not being reported by male heads of households who act as respondents and who choose to withhold information for instance if a pregnancy occurred out of wedlock or if the child died soon after birth without being named. As the neonatal period is short, a neonate who is born and subsequently dies in the period between two rounds of fieldwork may be entirely missed if the pregnancy was not recorded and the death is not revealed. The earlier the neonatal death the more likely it is to be missed. As the estimates in the DHS and HDSS are similar, it is likely that early neonatal deaths are missed by both data collection methods.

Both the DHS and HDSS showed that child mortality was the major contributor of under-five deaths until recently. By 2011 however, neonatal mortality had gained prominence. High child mortality is consistent with other sub-Saharan Africa estimates as infections particularly pneumonia and sepsis/ meningitis in those aged 1 to 4 years remain among the leading causes of under-five deaths in SSA [30].

Our assessment that the MICS can be used to measure the national trend in infant and child mortality implies that under-five mortality in The Gambia dropped from >200/1000 livebirths in the 1980s to 109/1000 livebirths in mid-2004. Although DHS estimates for earlier periods implied under-reporting of dead children by older women, for the most recent five-year period, the DHS estimates agreed well with the census and MICS reports of under-five mortality. According to these estimates, by 2011–12, a further drop in under-five mortality to 54/1000 live births occurred, and neonatal deaths comprised 40% of under-five mortality. As child mortality levels drop, neonatal mortality gains greater significance in under-five mortality [31–33].

In The Gambia, several targets for the MDGs were proposed depending on what estimate was adopted of under-five mortality in 1990. They ranged from 45–67.5/1000 livebirths [34, 35]. As our estimates place the U5MR at 135/1000 livebirths for the year 1990, the 45/1000 target for under-five mortality seems most legitimate. Based on this, The Gambia almost achieved the fourth MDG, attaining a 60% reduction, compared to the targeted 67% reduction, in under-five mortality. This decline has been attributed to proper prevention and treatment of malaria, appropriate and timely case management of childhood illnesses and immunization where national measles coverage reached 95% by 2013 [36, 37].

The 2012–20 Gambian National Health Policy and 2014–20 Strategic Plans aims to introduce free maternal and child health care, to improve doctor-to-patient ratios and increase access to insecticide-treated bed nets to further improve child survival [38, 39]. These reports acknowledge that the bulk of under-five mortality in The Gambia consists of neonatal deaths and several interventions are mentioned that relate to reduction of neonatal deaths including improvement in antenatal care through incentivization, skilled birth attendance, integrated management of neonatal illness and improved contraceptive use [38, 39].

## Conclusion

The first Gambian DHS for the most recent three to five years on infant and child mortality were of better quality than data about the early births of older women. The availability of disaggregated estimates of under-five mortality from the DHS reveals that The Gambia has experienced a large decline in both under-five mortality and, more recently, neonatal mortality. Neonatal mortality now makes a substantial contribution to under-five deaths. Based on the DHS estimates of neonatal and under-five mortality for the period surrounding 2010 (average rates for 2009–2012) of 23 and 56/1000 livebirths respectively, The Gambia will need to halve both these rates over a twenty-year period if it is to achieve the SDG goals of reducing neonatal mortality to 12/1000 live births and under-five mortality rate to 25/1000 livebirths by 2030. This may entail initiation of essential new-born care packages to improve neonatal survival on a country-wide basis, whilst maintaining or scaling up coverage of successful interventions that resulted in sustained decline in the other components of under-five mortality.

## Supporting information

S1 File Supplementary table_Rerimoi. Data quality assessment for direct estimation of mortality & Indirect mortality estimates.
(DOCX)

S2 File AnonymizedBHDSS.dta. (DTA)
