# Supplementary material for: Under-five mortality in The Gambia: Comparison of the results of the first demographic and health survey with those from existing inquiries
Source: PLoS One. 2019 Jul 23;14(7):e0219919. doi: 10.1371/journal.pone.0219919 (PMC6650053; doi:10.1371/journal.pone.0219919)
Supplement: S1 File — Data quality assessment for direct estimation of mortality & Indirect mortality estimates. (DOCX) [file pone.0219919.s001.docx]

**Supplementary Figures and Tables:** **Under-five mortality in The Gambia: Comparison of the results of the first Demographic and Health Survey with those from existing inquiries**

**Data Quality graphs**

***Data quality assessment of data for direct estimation of mortality***

Age heaping and date preference were assessed for both DHS and HDSS data. The ratios of under-five mortality indicators for DHS and HDSS data were also calculated. The age-heaping index for the neonatal period in the DHS was 1.15, while those for regions ranged from 0.85-1.71. For the HDSS, age heaping in the neonatal period was 0.90 and 0.94 for Basse and Farafenni respectively.

**Table 1: Age heaping indices for the neonatal period - Gambia 2003-12**

| Neonatal age heaping | Gambia DHS | Kerewan DHS | Basse DHS | Basse HDSS | Farafenni HDSS |
| --- | --- | --- | --- | --- | --- |
| day7 | 32 | 7 | 4 | 19 | 21 |
| day5-9 | 139 | 20 | 24 | 105 | 112 |
| fifth of 5-9 | 27.8186 | 4.083 | 4.7166 | 21 | 22.4 |
| heaping index | 1.15031 | 1.714426 | 0.848069 | 0.904762 | 0.9375 |
|  |  |  |  |  |  |

In Figures 1 and 2, the age at death in DHS data indicates that more deaths occurred in the first week of life, mostly on the first day. Notably, there are very few neonatal deaths reported in the late neonatal period, and those that are recorded correspond to 14 days (2 weeks) and 21 days (3 weeks).

**Figure 1: Age at death in days at the national level – 2013 Gambia DHS**

**Figure 2: Age at death in days Basse DHS and Kerewan DHS regions**

There were no missing dates in either the Basse or Farafenni HDSS. In the Basse HDSS, the 1^st^ was the most frequently recorded date of birth and death as shown for deaths in figure 3, requiring a random imputation of day of birth and death with preservation of month and year of birth as indicated in the Figures 3 and 4.

**Figure 3: Unadjusted dates of deaths in Farafenni and Basse HDSS**

**Figure 4: Adjusted date heaping in Basse HDSS**

Regarding the ratios of under-five mortality indicators, these were to be compared to the expected sub-Saharan Africa ratios of NMR: IMR of 0∙51 and NMR: U5MR of 0∙34. This comparison was restricted to 2007 to 2012 based on availability of Basse HDSS data, and are presented in Figures 5 and 6 respectively. The values reported in this section are the average ratios for the period of analysis.

For the HDSS, NMR: IMR in Basse was 0.38 and in Farafenni 0.46. The NMR: U5MR in Basse HDSS was 0.31 and in Farafenni 0.23. In the DHS, at national level NMR: IMR was 0.6, in both Basse and Kerewan 0.7. NMR: U5MR was 0.43 at national level, 0.45 in Basse and 0.6 in Kerewan.

Farafenni HDSS most closely approximated the expected NMR: IMR, and Basse HDSS the NMR: U5MR. The DHS ratios over time had more fluctuations, while the HDSS estimates were more stable as would be expected.

**Figure 5: Neonatal to infant mortality ratios for DHS and HDSS data**

**Figure 6: Neonatal to under-five mortality ratios for DHS and HDSS data**

**Table 2: Indirect Estimation of under-five mortality in The Gambia**

| **Region** | **year** | **u5mr** | **estimation** | **Source** |
| --- | --- | --- | --- | --- |
| Basse | 1998 | 217 | Indirect | MICS 2000 |
| Basse | 1996 | 244 | Indirect | MICS 2000 |
| Basse | 1993 | 259 | Indirect | MICS 2000 |
| Basse | 1991 | 252 | Indirect | MICS 2000 |
| Basse | 1988 | 284 | Indirect | MICS 2000 |
| Basse | 1985 | 341 | Indirect | MICS 2000 |
| Basse | 2003 | 116 | Indirect | MICS 2005 |
| Basse | 2002 | 196 | Indirect | MICS 2005 |
| Basse | 2000 | 176 | Indirect | MICS 2005 |
| Basse | 1998 | 225 | Indirect | MICS 2005 |
| Basse | 1996 | 249 | Indirect | MICS 2005 |
| Basse | 1993 | 259 | Indirect | MICS 2005 |
| Basse | 2008 | 95 | Indirect | MICS 2010 |
| Basse | 2006 | 148 | Indirect | MICS 2010 |
| Basse | 2003 | 135 | Indirect | MICS 2010 |
| Basse | 2001 | 199 | Indirect | MICS 2010 |
| Basse | 1998 | 176 | Indirect | MICS 2010 |
| Basse | 1995 | 196 | Indirect | MICS 2010 |
| Basse | 2001 | 112 | Indirect | Census 2003 |
| Basse | 1999 | 116 | Indirect | Census 2003 |
| Basse | 1997 | 134 | Indirect | Census 2003 |
| Basse | 1994 | 150 | Indirect | Census 2003 |
| Basse | 1991 | 170 | Indirect | Census 2003 |
| Basse | 1988 | 170 | Indirect | Census 2003 |
| Basse | 2011 | 56 | Indirect | Census 2013 |
| Basse | 2009 | 59 | Indirect | Census 2013 |
| Basse | 2006 | 70 | Indirect | Census 2013 |
| Basse | 2004 | 77 | Indirect | Census 2013 |
| Basse | 2001 | 94 | Indirect | Census 2013 |
| Basse | 1998 | 101 | Indirect | Census 2013 |
| Basse | 2011 | 96 | Indirect | DHS 2013 |
| Basse | 2009 | 89 | Indirect | DHS 2013 |
| Basse | 2007 | 102 | Indirect | DHS 2013 |
| Basse | 2004 | 137 | Indirect | DHS 2013 |
| Basse | 2002 | 112 | Indirect | DHS 2013 |
| Basse | 1999 | 155 | Indirect | DHS 2013 |
| Kerewan | 1997 | 116 | Indirect | MICS 2000 |
| Kerewan | 1996 | 146 | Indirect | MICS 2000 |
| Kerewan | 1994 | 152 | Indirect | MICS 2000 |
| Kerewan | 1992 | 154 | Indirect | MICS 2000 |
| Kerewan | 1990 | 171 | Indirect | MICS 2000 |
| Kerewan | 1987 | 140 | Indirect | MICS 2000 |
| Kerewan | 2004 | 122 | Indirect | MICS 2005 |
| Kerewan | 2002 | 118 | Indirect | MICS 2005 |
| Kerewan | 1999 | 136 | Indirect | MICS 2005 |
| Kerewan | 1997 | 128 | Indirect | MICS 2005 |
| Kerewan | 1994 | 123 | Indirect | MICS 2005 |
| Kerewan | 1991 | 183 | Indirect | MICS 2005 |
| Kerewan | 2008 | 67 | Indirect | MICS 2010 |
| Kerewan | 2006 | 93 | Indirect | MICS 2010 |
| Kerewan | 2003 | 111 | Indirect | MICS 2010 |
| Kerewan | 2000 | 134 | Indirect | MICS 2010 |
| Kerewan | 1997 | 143 | Indirect | MICS 2010 |
| Kerewan | 1994 | 148 | Indirect | MICS 2010 |
| Kerewan | 2001 | 112 | Indirect | Census 2003 |
| Kerewan | 1999 | 103 | Indirect | Census 2003 |
| Kerewan | 1997 | 122 | Indirect | Census 2003 |
| Kerewan | 1995 | 138 | Indirect | Census 2003 |
| Kerewan | 1992 | 164 | Indirect | Census 2003 |
| Kerewan | 1989 | 166 | Indirect | Census 2003 |
| Kerewan | 2011 | 57 | Indirect | Census 2013 |
| Kerewan | 2009 | 65 | Indirect | Census 2013 |
| Kerewan | 2007 | 81 | Indirect | Census 2013 |
| Kerewan | 2005 | 87 | Indirect | Census 2013 |
| Kerewan | 2002 | 114 | Indirect | Census 2013 |
| Kerewan | 1999 | 121 | Indirect | Census 2013 |
| Kerewan | 2011 | 48 | Indirect | DHS 2013 |
| Kerewan | 2009 | 59 | Indirect | DHS 2013 |
| Kerewan | 2007 | 67 | Indirect | DHS 2013 |
| Kerewan | 2005 | 69 | Indirect | DHS 2013 |
| Kerewan | 2002 | 95 | Indirect | DHS 2013 |
| Kerewan | 1999 | 120 | Indirect | DHS 2013 |
| Gambia | 1998 | 140 | Indirect | MICS 2000 |
| Gambia | 1996 | 166 | Indirect | MICS 2000 |
| Gambia | 1994 | 161 | Indirect | MICS 2000 |
| Gambia | 1992 | 170 | Indirect | MICS 2000 |
| Gambia | 1989 | 188 | Indirect | MICS 2000 |
| Gambia | 1986 | 218 | Indirect | MICS 2000 |
| Gambia | 2004 | 130 | Indirect | MICS 2005 |
| Gambia | 2002 | 130 | Indirect | MICS 2005 |
| Gambia | 2000 | 133 | Indirect | MICS 2005 |
| Gambia | 1997 | 139 | Indirect | MICS 2005 |
| Gambia | 1995 | 161 | Indirect | MICS 2005 |
| Gambia | 1992 | 187 | Indirect | MICS 2005 |
| Gambia | 2008 | 72 | Indirect | MICS 2010 |
| Gambia | 2006 | 97 | Indirect | MICS 2010 |
| Gambia | 2004 | 123 | Indirect | MICS 2010 |
| Gambia | 2002 | 136 | Indirect | MICS 2010 |
| Gambia | 1999 | 142 | Indirect | MICS 2010 |
| Gambia | 1996 | 155 | Indirect | MICS 2010 |
| Gambia | 2011 | 57 | Indirect | Census 2013 |
| Gambia | 2009 | 57 | Indirect | Census 2013 |
| Gambia | 2007 | 64 | Indirect | Census 2013 |
| Gambia | 2005 | 69 | Indirect | Census 2013 |
| Gambia | 2002 | 84 | Indirect | Census 2013 |
| Gambia | 1999 | 90 | Indirect | Census 2013 |
| Gambia | 2001 | 102 | Indirect | Census 2003 |
| Gambia | 1999 | 101 | Indirect | Census 2003 |
| Gambia | 1997 | 113 | Indirect | Census 2003 |
| Gambia | 1995 | 124 | Indirect | Census 2003 |
| Gambia | 1992 | 150 | Indirect | Census 2003 |
| Gambia | 1989 | 149 | Indirect | Census 2003 |
| Gambia | 2011 | 77 | Indirect | DHS 2013 |
| Gambia | 2009 | 62 | Indirect | DHS 2013 |
| Gambia | 2007 | 73 | Indirect | DHS 2013 |
| Gambia | 2005 | 84 | Indirect | DHS 2013 |
| Gambia | 2002 | 96 | Indirect | DHS 2013 |
| Gambia | 1999 | 108 | Indirect | DHS 2013 |

**Table 3: Direct estimation of under-five mortality in The Gambia**

| **Region** | **year** | **u5mr** | **95% CI** | **estimation** | **Source** |
| --- | --- | --- | --- | --- | --- |
| Gambia | 2003 | 93.7 | 73.7-113.8 | Direct | DHS 2013 |
| Gambia | 2004 | 82.6 | 64.2-100.9 | Direct | DHS 2013 |
| Gambia | 2005 | 92.8 | 71.9-113.7 | Direct | DHS 2013 |
| Gambia | 2006 | 90.7 | 69.7-111.6 | Direct | DHS 2013 |
| Gambia | 2007 | 76.4 | 60.3-92.6 | Direct | DHS 2013 |
| Gambia | 2008 | 67.0 | 49.9-84.1 | Direct | DHS 2013 |
| Gambia | 2009 | 71.0 | 52.6-89.5 | Direct | DHS 2013 |
| Gambia | 2010 | 61.1 | 47.1-75.1 | Direct | DHS 2013 |
| Gambia | 2011 | 50.9 | 38.4-63.4 | Direct | DHS 2013 |
| Gambia | 2012 | 63.8 | 45.8-81.8 | Direct | DHS 2013 |
| Basse | 2003 | 99.0 | 56.8-141.2 | Direct | DHS 2013 |
| Basse | 2004 | 124.7 | 74.1-175.3 | Direct | DHS 2013 |
| Basse | 2005 | 163.6 | 88.4-238.9 | Direct | DHS 2013 |
| Basse | 2006 | 168.9 | 90.4-247.4 | Direct | DHS 2013 |
| Basse | 2007 | 111.4 | 67.4-155.3 | Direct | DHS 2013 |
| Basse | 2008 | 128.0 | 59.4-196.6 | Direct | DHS 2013 |
| Basse | 2009 | 135.0 | 86.5-183.6 | Direct | DHS 2013 |
| Basse | 2010 | 94.9 | 59.8-129.9 | Direct | DHS 2013 |
| Basse | 2011 | 63.0 | 33.0-93.0 | Direct | DHS 2013 |
| Basse | 2012 | 67.5 | 36.8-98.2 | Direct | DHS 2013 |
| Kerewan | 2003 | 116 | 56.6-175.5 | Direct | DHS 2013 |
| Kerewan | 2004 | 101.9 | 55.9-147.9 | Direct | DHS 2013 |
| Kerewan | 2005 | 70.1 | 38.1-102.2 | Direct | DHS 2013 |
| Kerewan | 2006 | 94.0 | 53.7-134.3 | Direct | DHS 2013 |
| Kerewan | 2007 | 68.7 | 37.9-99.4 | Direct | DHS 2013 |
| Kerewan | 2008 | 33.3 | 11.8-54.9 | Direct | DHS 2013 |
| Kerewan | 2009 | 42.1 | 18.9-65.3 | Direct | DHS 2013 |
| Kerewan | 2010 | 56.7 | 20.0-93.4 | Direct | DHS 2013 |
| Kerewan | 2011 | 40.6 | 17.2-64.1 | Direct | DHS 2013 |
| Kerewan | 2012 | 19.3 | 3.4-35.2 | Direct | DHS 2013 |
| Farafenni | 1990 | 143.8 | 118.6- 173.9 | Direct | HDSS |
| Farafenni | 1991 | 190.3 | 162.4-222.3 | Direct | HDSS |
| Farafenni | 1992 | 162.8 | 136.8-193.2 | Direct | HDSS |
| Farafenni | 1993 | 233.5 | 203.6-267.1 | Direct | HDSS |
| Farafenni | 1994 | 187.8 | 159.7-220.2 | Direct | HDSS |
| Farafenni | 1995 | 208.9 | 179.3-242.6 | Direct | HDSS |
| Farafenni | 1996 | 137.3 | 112.4-167.3 | Direct | HDSS |
| Farafenni | 1997 | 122.4 | 98.3-152.0 | Direct | HDSS |
| Farafenni | 1998 | 165.0 | 138.5-195.9 | Direct | HDSS |
| Farafenni | 1999 | 199.4 | 171.0-231.8 | Direct | HDSS |
| Farafenni | 2000 | 164.6 | 138.6-194.8 | Direct | HDSS |
| Farafenni | 2001 | 125.2 | 102.2-152.9 | Direct | HDSS |
| Farafenni | 2002 | 100.1 | 83.3-120.0 | Direct | HDSS |
| Farafenni | 2003 | 80.9 | 68.2-96.0 | Direct | HDSS |
| Farafenni | 2004 | 69.7 | 58.1-83.6 | Direct | HDSS |
| Farafenni | 2005 | 62.4 | 51.6-75.3 | Direct | HDSS |
| Farafenni | 2006 | 50.5 | 40.9-62.4 | Direct | HDSS |
| Farafenni | 2007 | 57.1 | 47.1-69.0 | Direct | HDSS |
| Farafenni | 2008 | 48.5 | 39.5-59.5 | Direct | HDSS |
| Farafenni | 2009 | 30.1 | 23.2-39.2 | Direct | HDSS |
| Farafenni | 2010 | 33.4 | 26.0-42.8 | Direct | HDSS |
| Farafenni | 2011 | 40.1 | 32.0-50.2 | Direct | HDSS |
| Farafenni | 2012 | 50.8 | 41.9-61.5 | Direct | HDSS |
| Basse | 2009 | 63.3 | 57.5-69.5 | Direct | HDSS |
| Basse | 2010 | 76.9 | 70.8-83.5 | Direct | HDSS |
| Basse | 2011 | 63.9 | 58.4-69.9 | Direct | HDSS |
| Basse | 2012 | 55.2 | 50.2-60.7 | Direct | HDSS |
